# Ultra-Low Dose Cytokines in Rheumatoid Arthritis, Three Birds with One Stone as the Rationale of the 2LARTH^®^ Micro-Immunotherapy Treatment

**DOI:** 10.3390/ijms22136717

**Published:** 2021-06-23

**Authors:** Camille Jacques, Ilaria Floris, Béatrice Lejeune

**Affiliations:** Preclinical Research Department, Labo’Life France, 1 Rue François Bruneau, 44000 Nantes, France; ilaria.floris@labolife.com (I.F.); beatrice.lejeune@labolife.com (B.L.)

**Keywords:** rheumatoid arthritis, TNF-α, IL-1β, ultra-low doses, micro-immunotherapy, anti-inflammatory medicines, hormesis, chronic inflammation, inflammatory cytokines

## Abstract

Tumor necrosis factor-α (TNF-α) and interleukin-1β (IL-1β) are two cytokines involved in the perpetuation of the chronic inflammation state characterizing rheumatoid arthritis (RA). Significant advances in the treatment of this pathology have been made over the past ten years, partially through the development of anti-TNF and anti-IL-1 therapies. However, major side effects still persist and new alternative therapies should be considered. The formulation of the micro-immunotherapy medicine (MIM) 2LARTH^®^ uses ultra-low doses (ULD) of TNF-α, IL-1β, and IL-2, in association with other immune factors, to gently restore the body’s homeostasis. The first part of this review aims at delineating the pivotal roles played by IL-1β and TNF-α in RA physiopathology, leading to the development of anti-TNF and anti-IL-1 therapeutic agents. In a second part, an emphasis will be made on explaining the rationale of using multiple therapeutic targets, including both IL-1β and TNF-α in 2LARTH^®^ medicine. Particular attention will be paid to the ULD of those two main pro-inflammatory factors in order to counteract their overexpression through the lens of their molecular implication in RA pathogenesis.

## 1. An Introduction to Rheumatoid Arthritis and Micro-Immunotherapy

Rheumatoid arthritis (RA) is a widespread systemic autoimmune disease characterized by chronic inflammation of the articular membrane, synovial hyperplasia, and progressive degradation of cartilage and bone, leading to joint destruction. Patients with RA usually experience joint pain, swelling, tenderness, and stiffness, especially in the morning, and they also have to cope with fatigue and depression as the disease represents high personal and social burden [1]. Studies conducted in Northern European and North American areas evaluated RA prevalence at about 0.5–1% [2], whereas it appeared less frequently in low- and middle-income countries, where it is gauged at 0.4% and 0.37% in Southeast Asian and in Eastern Mediterranean regions, respectively [3]. Epidemiologic data reported that RA susceptibility increased with age and that sex was also an important factor to take into account. This debilitating disease affects women at a rate double that in men, and in more aggressive forms [4]. Even if the etiology of RA is still unknown, 50–60% of the cases are hereditary and the human leukocyte antigen (HLA) locus is involved in at least 30% of the overall genetic risk.

As research progresses in delineating the drivers of this pathology, it appears that multiple immune factors, all integrated in a complex network and in a particular temporal frame, are involved in the onset and the progression of the disease. For instance, it is now well-established that numerous pro-inflammatory cytokines and growth factors perpetuate the chronic inflammation state of RA, in particular, interleukin-1β (IL-1β) and tumor necrosis factor-α (TNF-α). Cytokines are typically secreted by immune cells in order to orchestrate diverse cellular functions, such as cell differentiation, activation, migration, survival or proliferation in a paracrine or autocrine manner. As they display pleiotropic effects, can either act synergistically or share redundancy of actions one with the others, a fine-tuned regulation of their interplay is of paramount interest in the proper immune system maintenance and the body’s homeostasis. Their perturbation in the development of RA is well-documented, some evidence even showing that a subset of cytokines is already imbalanced before the clinical onset of the disease [5].

Significant advances in the treatment of this pathology have been made over the past ten years and the clinical arsenal mainly encompasses the so-called disease-modifying anti-rheumatic drugs (DMARDs) including methotrexate, sulfasalazine, hydroxychloroquine and leflunomide, the janus-activated kinase 3 (JAK3) inhibitor tofacitinib, glucocorticoids and biologic agents such as infliximab, etanercept, adalimumab, golimumab, and certolizumab pegol. All of them are used in both early and established RA (< or >6 months), depending on patients’ values, preferences and comorbidities [6,7]. Regarding the pivotal role played by the resident/immune cells hosted in the synovial tissue in the pathogenesis of the disease, cell-targeted therapies have also been developed. Unfortunately, the parenteral administration required for those therapies is still a limiting factor towards their use. Moreover, some unavoidable issues still persist: (1) most of the therapeutics can only alleviate symptoms without intrinsically treating the root of the problem, ultimately leading to an incomplete remission; (2) regarding the length and the costs of the treatment regimens, some patients struggle with compliance, and (3) side effects are still substantial.

In line with those prerequisites, novel treatments—sequentially able to modulate the expression of multiple immune players over time, as the stage of the pathology evolves—would be of great interest. They would indeed allow to re-establish the body’s homeostasis, both at a systemic level and within the injured joint niches. For this purpose, the traditional therapeutic strategy of the micro-immunotherapy medicine (MIM) 2LARTH^®^ has been developed to attenuate the symptoms of rheumatic diseases including RA, within a holistic immunomodulatory approach. Micro-immunotherapy (MI) is a pioneering modulative therapeutic approach that can be employed alone or in association with other therapies, providing clinical benefits while reducing side effects. This strategy combines the use of several immune system mediators such as cytokines, growth factors, hormones, nucleic acids and specific nucleic acids (SNA^®^), in order to orchestrate both the innate and the adaptive/acquired immune responses. SNA^®^ (European Patent: EP0670164B1) consist of single-stranded DNA molecules of 16–44 bases, specifically designed to target one gene or more, based on sequence complementarity. The active ingredients of the MI medicines are prepared according to repetition series of a two-step process called “sequential kinetic process”, consisting of (1) 1/100 dilution of the above-mentioned immune mediator, followed by (2) calibrated vertical shaking, reproduced until the desired dilution is reached. One step of this manufacturing process thus defines 1 CH (centesimal Hahnemannian) dilution, in accordance with the European Pharmacopeia monographs 1038 and 2371, current edition. Low doses (LD) range from 1 CH to 7 CH and are used in MI to boost the immune system, activate or increase protein expression, while ultra-low doses (ULD) aim at modulating (from 8 to 12 CH) and lowering (higher than 12 CH) the expression of protein(s) with upregulated levels. LD and ULD are finally impregnated on lactose-sucrose pillules. MI medicines are based on the principle that a given immune regulator exerts either stimulatory effects when used at LD or displays modulatory/inhibitory features when prepared at ULD.

The first part of this review aims at delineating the pivotal roles played by IL-1β and TNF-α in RA physiopathology, leading to the development of anti-TNF and anti-IL-1 therapeutic agents. In a second part, an emphasis will be made on explaining the rationale of using multiple therapeutic targets, including both IL-1β and TNF-α in 2LARTH^®^ medicine. Particular attention will be paid to the ULD of those two main pro-inflammatory factors in order to counteract their overexpression, through the lens of their molecular implication in RA pathogenesis.

## 2. IL-1β and TNF-α in the Physiopathology of RA, the Bad and the Ugly

Rheumatoid arthritis, as a form of inflammatory pathology, could be qualified as both (1) a systemic and (2) a localized disease, as the systemic serum cytokine levels are affected over the course of the pathology progression, and also because cytokines crosstalk is intrinsically involved in a complex process that transforms the joint into a site of insistent inflammation-promoting tissue necrosis and degradation. The synovial lining, in normal condition, is mainly made of fibroblastic cells, the so-called “synoviocytes” along with macrophages. During the course of RA onset, the synovial delineation expands dramatically, as the synoviocytes’ growth becomes anarchic, finally reaching a point where the synovial membrane is so thick that the joint is completely embedded into a “pannus” structure. In this context, the synovial fibroblasts produce mediators actively participating in the cartilage and joints destruction [8]. Immune cells like B cells, T cells, macrophages, and neutrophils progressively infiltrate the synovial fluids and their crosstalk perpetuates the production of pro-inflammatory cytokines and mediators, such as IL-1β, IL-2, and TNF-α.

### 2.1. IL-1β, the Bad

**Figure 1:**
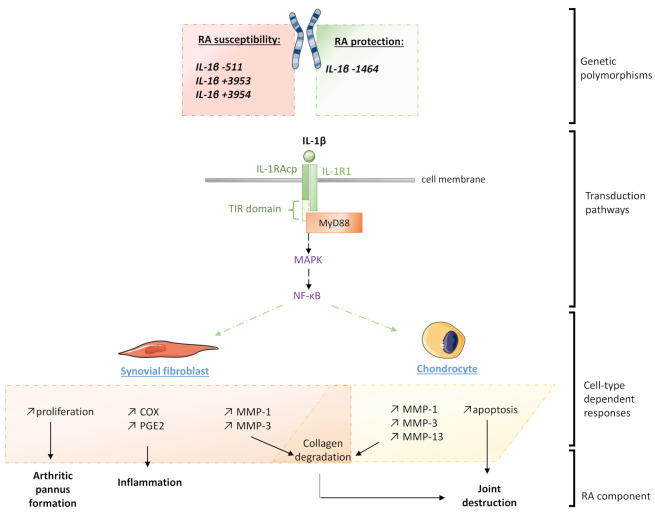
Schematic representation of the different levels of IL-1β’s implication in the pathogenesis of rheumatoid arthritis (RA). IL-1R1: Interleukin-1 type-I receptor; IL-1RAcP: IL-1 receptor accessory protein; MAPK: Mitogen-activated protein kinase; MMP: Matrix metalloproteinase; MyD88: Myeloid and differentiation primary response 88; NF-κB: Nuclear factor-κB; PGE2: Prostaglandin E2; TIR: Toll-IL-1 receptor.

Although the IL-1 family originally only encompasses IL-1α and IL-1β, eleven members are currently listed [9]. These cytokines have the ability to bind several kinds of receptors, encoded by nine distinct genes, including coreceptors, decoy receptors, binding proteins and inhibitory receptors. The binding mechanism of both IL-1α and IL-1β is a two-step process starting by the cytokine attachment to the ligand-binding chain, termed type-I receptor (IL-1RI), followed by the recruitment of the coreceptor chain, named the accessory-protein (IL-1RAcP). The transduction of the signal is then mediated by the adaptor protein myeloid and differentiation primary response 88 (MyD88), recruited to the Toll-IL-1 receptor (TIR) domain of IL-1RAcP (Figure 1). A cascade of phosphorylation and ubiquitination events then results in the activation of nuclear factor-κB (NF-κB), leading to the expression of pro-inflammatory genes in the stimulated cells [10]. As IL-1β is mainly produced by myeloid cells upon inflammatory stimulation, this cytokine also plays a major role in immunity and hematopoiesis.

In the RA context, the deleterious effects of the IL-1β within the articular niche are broadly reported, as this cytokine induces cellular responses in the major cells constituting the joint microenvironment (Figure 1). For instance, synovial fibroblasts isolated from RA patients undergoing knee arthroplasty display an increased proliferative capacity in the presence of IL-1β [11]. Stimulation with cytokines at only 1 ng/mL was also shown to induce the expression of matrix metalloproteinases-1 and -3 (MMP-1 and MMP-3), cyclooxygenase-2 (COX-2), and prostaglandin E2 (PGE2), both at RNA and protein levels, through the activation of the MAPKs (mitogen-activated protein kinases) and the NF-κB pathways. Kobayashi et al. confirmed those effects within an osteoarthritis cartilage explant model in which they used the IL-1RA antagonist anakinra to block the IL-1β biologic activity, thus demonstrating the implication of this cytokine in the production of MMPs (MMP-1, MMP-3 and MMP-13) [12]. Moreover, IL-1β also directly acts on chondrocytes in decreasing their viability, as well as inducing their apoptosis, as Chen et al. reported in their in vitro experiments [13]. Interestingly, a distinct pro-inflammatory macrophage subtype characterized by a CD14^+^ IL-1β^+^ phenotype was shown to be obviously expanded in RA compared to the non-inflammatory osteoarthritis disease [14].

From a genetic standpoint, some associations have been made between IL-1β mutations and the susceptibility of RA occurrence depending on the population analyzed (Figure 1). A study conducted in Algerian people reported that the (T/T) polymorphism of IL-1B –511 was more frequent in RA patients with anti-citrullinated peptide antibodies (ACPA), compared with the negative ACPA group [15]. This association was confirmed by another study conducted in Indian RA patients, where this polymorphism would tend to be associated with elevated serum anti-CCP (cyclic citrullinated peptides) and high IL-1β levels, both known as inflammatory markers in this context [16]. Furthermore, an ethnicity-specific meta-analysis suggested that the IL-1B −511 C/T polymorphism was associated with RA susceptibility in Caucasians, whereas the IL-1B +3953 C/T polymorphism was associated with susceptibility to RA in Caucasian and in Asian populations [17]. Other studies found that IL-1B +3954 polymorphism was significantly associated with the risk of RA in the overall population and in Asian people [18,19]. Furthermore, an analysis conducted in the Japanese population revealed that individuals affected with both RA and periodontitis may display different distributions of IL-1B +3954 genotypes than healthy controls and subjects with periodontitis only [20]. A British study reported that the IL-1B −1464 C/G allele was less common in RA patients than in healthy individuals, thus suggesting a protective effect of this variant, whereas the statistically significant association between IL-1B −511 A/G and RA was also delineated [21].

### 2.2. TNF-α, the Ugly

**Figure 2:**
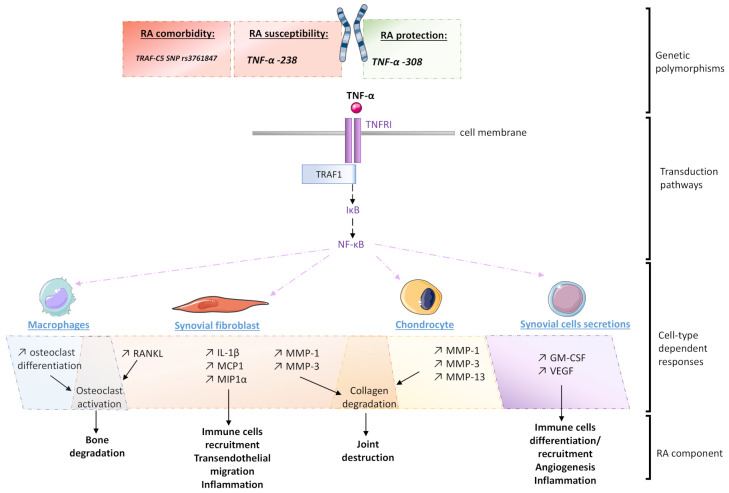
Schematic representation of the different levels of TNF-α’s implication in the pathogenesis of rheumatoid arthritis (RA): IL-1β: Interleukin-1β; IκB: Inhibitor of nuclear factor-κB; GM-CSF: Granulocyte-macrophage colony-stimulating factor; MAPK: Mitogen-activated protein kinase; MCP1: Monocyte chemoattractant protein-1; MIP1α: Macrophage inflammatory protein-1 alpha; MMP: Matrix metalloproteinase; NF-κB: Nuclear factor-κB; RANKL: Receptor activator of nuclear factor-κB ligand; SNP: Single nucleotide polymorphism; TIR: Toll-IL-1 receptor; TNFRI: TNF receptor type 1; TRAF1: TNF receptor-associated factor; TRAF-C5: TNF receptor-associated factor 1 and complement component 5; VEGF: Vascular endothelial growth factor.

Isolated for the first time in 1975 by Carswell et al., in their endotoxin-mediated tumor necrosis experiments, the body of knowledge about TNF-α has tremendously grown ever since [22]. Many cell types are known to be TNF-α producers, including monocytes and macrophages. Once released, it binds to two different cell-surface receptors, namely, TNF receptor type 1 (TNFR1), also known as p55, which is ubiquitous, and TNFR2, also known as p75, whom the expression is more specific, including immune cells like Tregs for example [23]. As for the IL-1β, TNF-α activates NF-κB signaling by stimulating the proteolytic breakdown of its cytoplasmic inhibitor, IκB (inhibitor of nuclear factor-κB) (Figure 2). This pathway is known to elicit the expression of the TNF-α genes, further inducing various cellular responses including apoptosis, which occurs after the TNF-α binding to TNFR1, due to the presence of its C-terminal “death domain” [24]. TNF-α’s activity blockade through soluble PEGylated TNFR1 was shown to decrease the production of MMP-1, MMP-3 and MMP-13 in femoral condylar cartilage cells cultured from osteoarthritis patients [12]. TNF-α is a major factor of RA development within the synovial niche, as human synovial fibroblasts cultured in presence of TNF-α display increased expression of IL-1β, monocyte chemoattractant protein-1 (MCP1), macrophage inflammatory protein-1 alpha (MIP1α), MMP-1, MMP-3 and receptor activator of nuclear factor-κB ligand (RANKL), an osteoclastogenic cytokine, compared to control cells [25]. In line with these results, TNF-α also induces osteoclast differentiation in macrophages isolated from mouse bone marrow. Anti-TNF was also reported to reduce granulocyte-macrophage colony-stimulating factor (GM-CSF) and vascular endothelial growth factor (VEGF) both in vitro and in patients with RA [26,27].

As for the previously discussed IL-1β, the TNF-α genetic background is linked with RA development (Figure 2). For instance, a study reports that the TNF-α −238 G/A polymorphism contributes to the susceptibility risk to young-onset RA, whereas TNF-α −308 G/A variation may protect against the disease [28]. Interestingly, TNF-α −238 G/A polymorphism was not shown to be an RA susceptibility contributor in the Iranian population [29]. These discrepancies highlight the fact that even if genetics may set up a favorable cellular environment towards the spreading of RA, other regulation aspects should be taken into consideration. Regarding this example, the overall economic and social culture of a population may also be seen as an additional epigenetic layer governing RA occurrence. Moreover, the possible existence of TNF-α gene promoter variants acting as markers for RA severity and treatment response has been reported, reinforcing the role of this growth factor as a key immune player to target in this pathology [30]. Genetic comorbidities involving TNF-α pathway have also been reported, as it was found that RA patients bearing GG homozygosity at the TRAF1-C5 SNP rs3761847 were at a higher risk of death from cancer or sepsis [31]. Indeed, the TNF receptor-associated factor 1 (TRAF1) and complement component 5 (C5) is known to act as a scaffold protein complex and play a direct role in the downstream TNF-α signaling, further reinforcing the link between the TNF-α pathway and the immune escape associated with RA outcome and comorbidities.

## 3. RA and Cytokines: A TNF-α and IL-1β Crosstalk Modeled Both In Vitro and In Vivo

The synovial niche’s crosstalk in RA conditions was studied by Saeki et al., in their in vitro murine arthritis tissue-derived cells model [32]. They recently published that synovial macrophages cultured within conditioned-medium from synovial fibroblasts display changes in genic expression characterized by an upregulation in the expression of inflammatory markers such as Nos2, TNF-α, IL-1β and CD86. Additionally, these macrophages were reported to increase their TNF secretion. Moreover, TNF-α secretion was also found to be induced by DNA stimulation in the RA context, as CpG-rich DNA from blood plasma isolated from an RA patient was shown to stimulate the TLR9-MyD88-NF-kB signaling pathway when added to the culture medium of PBMCs (peripheral blood mononuclear cells) from healthy donors, resulting in a significant increase in the IL-6 and TNF-α secretion [33]. In their study, Schierbeck et al. reported that the TNF-α secretion induced in monocytes stimulated by LPS (lipopolysaccharide) + IFN-γ (interferon-γ) was significantly decreased when the cells were pretreated for one hour with the anti-TNF-α etanercept at either 2 or 8 µg/mL, whereas the anti-IL-1β anakinra (from 2.5 to 20 µg/mL) didn’t show any effect on the TNF-α secretion [34]. Furthermore, the gap junction mediator connexin-43 (Cx43) was shown to be involved in both gene expression and secretion of the TNF-α and IL-6 cytokines in a human RA synovial fibroblast model [35]. Matsuki et al. indeed showed that the siRNA-mediated Cx43 inhibition impaired the expression of these cytokines. They also delineated the existence of a retro-control loop, as the TNF-α stimulation of the cells markedly increased their own Cx43 expression. These data even strengthen the fact that TNF-α acts as a key player in the chronicity of the RA disease and its related inflammatory-vicious cycle, as its expression and secretion are induced by a plethora of factors on a broad number of cells from the synovial niche.

The increase of pro-inflammatory cytokines expression such as TNF-α, IL-1β, IL-6 and IL-17, inflammatory mediators like COX-2 and 5-LOX (5-lipoxygenase), as well as the reduction of anti-inflammatory markers such as IL-4 and IL-10 are the hallmark of synovial inflammation and cartilage damages and are extensively monitored parameters in in vivo RA models [36,37]. The CIA (collagen-induced arthritis) model is used as an industry-standard RA model in the evaluation of potential therapeutic agents as it allows for a deep chronic inflammatory process, partially mediated by the induced autoreactivity of T and B cells and reproduces the pannus development, the fibrin deposition, the joint inflammation and its osteo-articular damages characterizing the human disease. In addition, in this model, TNF-α, IL-1β, and IL-6 are also key players in the evolution of the pathology, from its early onset to the chronic phases [38]. In a CIA-induced mice model, it was found that the blockade of IL-1β, TNF-α or a combination of both cytokines delays the start of arthritis [39]. Furthermore, the authors reported that the anti-IL-1β treatment reduced the RA burden to a greater extent than the anti-TNF-α one, both clinically and histologically. Another study performing an in vivo TNF-α inhibition through adalimumab showed that the mRNA expression of IL-2 in the spleen of the treated mice was similar to the one of the healthy control group after daily subcutaneous injections of 1 mg/kg antibody [40]. Moreover, both the IL-1β and the TNF-α protein levels were reduced in the serum as well as in the spleens of the treated animals. In addition, the RANKL mRNA levels were also decreased in the spleens of the treated mice. The study reporting the benefits of using intra-articular injections of the anti-TNF infliximab, loaded onto thermosensitive hydrogel to decrease pro-inflammatory cytokines within the synovial fluids of a rabbit model of RA induced by ovalbumin and complete Freund’s adjuvant, is another example illustrating the interplay between TNF-α and IL-1β in vivo [41]. Indeed, two and six weeks after injections, the intra-articular levels of both TNF-α and IL-1β were significantly decreased, compared with the saline-treated control. Altogether, this evidence sustains the therapeutic strategies aiming to individually target these mediators and have already been developed in clinics, whereas they still have to translate into efficient cures devoid of unwanted adverse effects for the patients.

## 4. Anti-IL-1β and -TNF-α Therapies: Effects and Side Effects of the Conventional Allopathic Doses

**Figure 3:**
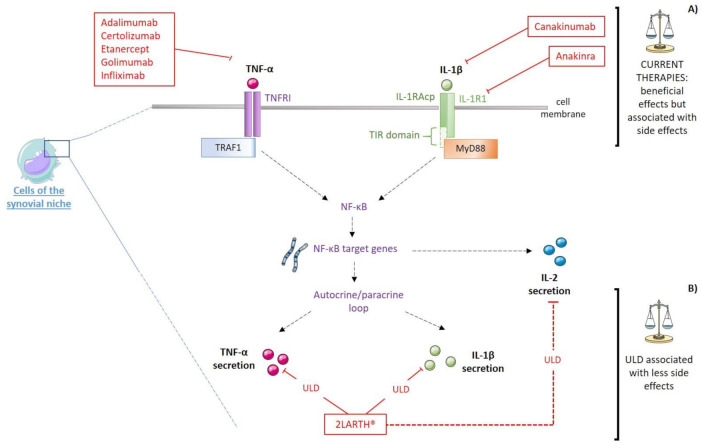
Schematic representation of (**A**) therapeutic targets of current treatments of RA and (**B**) the putative targets of 2LARTH^®^: IL-1R1: Interleukin-1 type-I receptor; IL-1RAcP: IL-1 receptor accessory protein; MAPK: Mitogen-activated protein kinase; MyD88: Myeloid and differentiation primary response 88; NF-κB: Nuclear factor-κB; TIR: Toll-IL-1 receptor; TNFRI: TNF receptor type 1; TRAF1: TNF receptor-associated factor 1; ULD: Ultra-low dose. Red plain lines represent inhibition mechanisms, red dotted lines represent modulatory mechanisms.

IL-1β antagonism has been performed in the context of RA through several options; either targeting the cytokine itself or its receptor. For instance, canakinumab (Ilaris^®^) is a humanized IgGκ monoclonal antibody against IL-1β, initially developed for the treatment of immune disorders (Figure 3A) [42]. In patients undergoing active RA despite being treated with methotrexate, the addition of subcutaneous injections of 150 mg canakinumab every four weeks over a 12-weeks period improved their therapeutic response regarding the global assessment of disease-related parameters [43]. Kinetic studies and modeling simulations used to extract the dose-response relationships confirmed that 150 mg every four weeks allowed for the capture of the majority of IL-1β, lowering it below an EC_50_ allowing to improve the ACR (American College of Rheumatology) scores in patients with RA [44]. Moreover, canakinumab has been considered as a good option for young patients with severe and/or refractory forms of IL-1-driven RA, in order to avoid joint deformation [45].

Anakinra is a human IL-1R antagonist currently approved for the treatment of RA, leading to significant improvements in disease symptoms and quality of life, regarding pain, Larsen radiographic scores and erythrocytes sedimentation rates, slowing down both radiologic manifestations of joint damages and bone erosion [46,47]. Furthermore, the treatment with this agent was shown to have beneficial effects on inflammatory and metabolic parameters, allowing to discontinue the concomitant use of glucocorticoids and anti-diabetic drugs in patients displaying both RA and type 2 diabetes [48]. Anakinra is mainly used as a second-line treatment in patients previously treated with a failing anti-TNF-α therapy or experiencing cancer or infectious disease such as *Mycobacterium tuberculosis* infections [49].

In parallel with the anti-IL-1β drugs, anti-TNF therapies aim at neutralizing TNF-α, therefore preventing it from exerting its pro-inflammatory role (Figure 3A). In RA, anti-TNF treatment was shown to reduce the production of pro-inflammatory cytokines, including IL-1 and IL-6, thus contributing to the disturbance of the crosstalk between those pathology drivers [50]. To date, several monoclonal antibodies have been developed and are approved by the FDA (Food and Drug Administration) in the United States for clinical use since 1998, such as infliximab, followed by adalimumab, certolizumab or golimumab. Adalimumab is a fully recombinant human monoclonal antibody, while infliximab is a chimeric monoclonal antibody made of about 75% of human-derived amino acids and 25% of mouse-derived amino acids. Certolizumab pegol is the only PEGylated TNF inhibitor, where the PEG (polyethylene glycol) attachment allows to increase its half-life in vivo, while it may also contribute to a better pharmacologic distribution into the inflamed arthritic tissues, compared with non-PEGylated antibodies [51]. Despite their effects in reducing the RA symptoms, those chimeric, human, or humanized antibodies are all associated with more or less severe liver injuries [52]. In addition, according to the European Medicines Agency, adalimumab is very frequently associated with infections and infestations, the more recurrent being the ones related to the respiratory tracts, sepsis, candidiasis and influenza [53]. Other side effects include infections of the skin and the urogenital tract; hematologic affections, like leukopenia and anemia; metabolism and nutrition defects, like lipids and hepatic enzymes increased levels; nervous system infections, like cephalea; gastrointestinal related disorders, including abdominal pain and nausea; musculo-skeletal pain, and injection-site reactions, like erythema. Anti-TNF drugs can also lead to the development of psoriasis-like skin lesions called paradoxical psoriasis, in about 2–5% of the treated patients, a condition, which, unfortunately, frequently requires the complete cessation of the treatment [54]. Moreover, Rotondo et al. suggested that pharmacologic immunosuppressive therapies such as anti-TNF could modify the immune response to Merkel cell Polyomavirus (MCPyV), thus undermining the outcome for positive MCPyV RA patients and increasing the probability of Merkel cell carcinomas (MCC) development, a rare but aggressive neuroendocrine tumor [55]. A Brazilian study also reported another TNF-α blocker adverse effect, as the use of such therapeutic agents was shown to be associated with high risk of active mycobacterial infections in patients with chronic inflammatory arthritis including RA even if they did not present any evidence of latent tuberculosis infection [56].

In addition, the TNF-α signalization blockade has also been done by inhibiting the TNF-α binding to its receptor through etanercept, a fully human soluble TNF receptor Fc fusion protein, where a dimer of the extracellular domains of human TNFR2 is fused to the Fc portion of human IgG1. The effectiveness of this drug has been established for the early administration from diagnosis of RA [57,58]. A phase IV study recently assessed the real-world safety and effectiveness of biosimilar etanercept in patients with RA, ankylosing spondylitis or psoriatic arthritis and receiving biosimilar etanercept injections, either 25 mg twice weekly or 50 mg once weekly [59]. The results reported that even if the HAQ (Health Assessment Questionnaire) scores decreased from 1.32 ± 0.77 at baseline to 0.81 ± 0.61 at 12 months in patients with RA and psoriatic arthritis (*p* < 0.01), some patients still reported adverse effects such as injection-site reactions, abdominal pain and upper respiratory tract infections. The European Medicines Agency reports that the more frequent side effects for this medicine are infections such as bronchitis, cystitis and cutaneous affections, as well as allergic reactions [60].

Interestingly, besides the above-mentioned unwanted side effects, a meta-analysis highlighted that TNF-α antagonists may have a beneficial effect on aortic stiffness, therefore related to cardiovascular risk, which RA patients are more prone to [61].

## 5. Predictive Markers… as a New Option towards Personalized Medicine

Along with the development of direct IL-1β and TNF-α inhibition as a therapeutic strategy, attempts were made to use those baseline cytokine levels as predictive biomarkers of RA or markers of treatment responses, allowing for a better stratification of the patients and their clinical care.

IL-1β, IL-2, and TNF-α are undetectable, or at very low levels in human circulation of healthy controls, often under the limit of detection (few pg/mL). Serum levels of IL-1β in healthy subjects were found to be about 3 pg/mL, IL-2 levels of about 14 pg/mL [62,63], and TNF-α levels of about 5 pg/mL [64]. While the circulating levels of those cytokines have been found sometimes higher in RA patients than control groups [64,65,66], the research is currently still trying to find out the applicability of cytokines as biomarkers in RA [67,68]. In addition, circulating TNF-α and IL-1β levels failed to predict response to rituximab, an anti-CD20 antibody [69]. RA patients with higher basal serum level of several pro-inflammatory cytokines, including IL-1β, IL-2, and TNF-α, were shown to better respond to tocilizumab, an anti-IL-6R treatment [70]. These data highlight the fact that the cytokine profile determination at the time of diagnosis could be used as a tool to orient the treatment strategy. Further in-depth studies are still needed in this area. In line with these results, it has recently been shown that a serum miRNA signature discriminated both RA patients and “at risk individuals” from healthy subjects [71]. The miRNA profiling included eight (miR-126-3p, let-7d-5p, miR-431-3p, miR-221-3p, miR-24-3p, miR130a-3p, miR-339-5p, let-7i-5p) upregulated and one (miR-17-5p) downregulated miRNAs. Interestingly, computational analysis revealed that their predictive targets could be involved in the regulation of inflammatory pathways, including NF-κB signaling, as well as TNF-α and IL-1β.

Regarding their ability to modulate multiple targets at once and to regulate pro-inflammatory pathways and cytokines including TNF-α and IL-1β in autoimmune diseases including RA, miRNAs have also been considered as interesting therapeutic agents on their own [72]. To our knowledge, no direct targeting of IL-1β or TNF-α by such miRNAs have been shown in RA models. Of note, the miR-140-5p has been shown to directly target the 3′UTR region of TNF-α, but this work has been done in a pulmonary arterial hypertension model [73]. Other authors have shown that the simultaneous intra-articular injections of this miRNA with the miR-140-3p ameliorates the RA features [74] and these results are corroborated by the fact that simultaneous overexpression of miR-140-5p and miR-146a in osteoarthritic chondrocytes reduced NF-κb phosphorylation, as well as the expression of TLR4, IL-1β, IL-6, and TNF-α [75]. As an in-depth study of the miRNAs involved in the regulation of these pathways would be worth an entire review, Table 1 provides a non-exhaustive list of some miRNAs which have been shown to be dysregulated in RA, or whom the preclinical data available ultimately lead to a modulation of the TNF-α and/or IL-1β cytokines expression.

Despite the above-mentioned advances in RA treatment and the hope given by a prospective serologic profiling, it is still estimated that about 20–40% of the patients are not good responders to anti-TNF therapies [80]. Such figures reinforce the fact that each therapeutic strategy should be adapted, personalized and open the application field for different cytokine-combining targeting strategies and dosages, such as Micro-Immunotherapy.

## 6. The Immunotherapy Based on Low Amount of Cytokines

Very few studies reported the use of cytokines at low amounts in clinics. The effect of a low amount IL-2 treatment (1 × 10^5^ IU/3 days) was assessed in vivo and was reported to reduce the severity of vascular and bone lesions in a CIA mice model, when intravenous injections were performed 14 days after the arthritis induction [81]. Moreover, authors showed that the percentage of pro-inflammatory cytokines secreted by spleen cells, such as IFN-γ, IL-17, and TNF-α was significantly reduced by the IL-2 injections. In addition, a prospective clinical study conducted in patients with mild to moderate forms of autoimmune disease, including RA, demonstrated the safety of an injection protocol consisting of a small amount of IL-2 (1 × 10^6^ IU/day) for five days, followed by fortnightly injections for six months [82]. Even if these concentrations are not obtained from centesimal Hahnemannian dilution protocols and a bona-fide sequential kinetic process, their safety for the patients let presume a promising future for the cytokine-based treatment in the context of RA. Regarding the pleiotropic effects of the cytokines and their implication in a broad spectrum of chronic systemic immune-mediated diseases, inhibiting the cell-secretion of such particular mediators through ultra-low delivery of those same exact mediators also seems appealing. Such approaches could be employed either as: (1) therapies on their own, (2) second-line treatments after a first therapy, or (3) scaffold strategies to set up a biological environment allowing a better outcome for future second-line conventional therapies. In the above mentioned (1) context, preliminary clinical, and preclinical studies highlighted the beneficial effects of 2LALERG^®^ as potential preventive MI medicine in patients suffering from allergic airway diseases [83,84]. While in a small number of patients, the double-blind versus placebo study showed that 2LALERG^®^, when taken two months before the pollinic season, has helped in the management of symptoms, it has worked as well as scaffold therapy (context 3) during the pollinic season, because patients decreased significantly their consumption of antihistamines and intranasal corticosteroids.

The potential effects of MI in the context (1) of chronic infections, such as high-risk human papillomavirus cervical lesions, showed that 2LPAPI^®^, alone, taken during six months, could induce and/or help in the regression of the cervical lesions, without side effects [85]. In the context (2), a tendency towards a reduced serum level of the cytokine RANTES (regulated upon activation, normal T cells expressed and secreted) was found in patients with systemic immune-mediated diseases undergoing fatty-degenerative osteolysis of the jawbone (FDOJ) surgery and treated with RANTES 27 CH, and no adverse effects were reported by the patients [86]. Surgical removal of FDOJ tissue, highly expressing RANTES, might not be sufficient to recreate the correct immunological environment. By using ULD of that same cytokine after surgery, MI aimed at re-establishing the homeostasis.

Moreover, in the (2) situation, an interesting study reported the positive effects of low dose sequential kinetic activation (SKA) cytokines/antibodies (IL-4, IL-10 and anti-IL-1) orally administered in RA patients who display a low disease activity (LDA) after having been treated with either anti-TNF-α biologic agents and/or DMARDs [87]. Indeed, even if the clinical results were obtained from a small cohort of 34 patients and are not significant, 10 fg/mL/day of the SKA cocktail of cytokines/antibodies allowed a rate of maintenance of LDA of 66.7% versus 42.1% in the DMARD treated group and no side effects were reported.

An in vitro study has been designed to assess the ULD (27 CH) of IL-1β and TNF-α abilities to decrease the TNF-α secretion of human LPS-exposed enriched monocytes after 24 h incubation periods [88]. The results showed a decrease of the TNF-α secretion when the lactose-sucrose pillules impregnated with the 27 CH cytokines, diluted at 11 and 22 mM, were compared with the vehicle-impregnated ones. Interestingly, these results were confirmed in PMA-differentiated THP-1 cells. These anti-inflammatory effects induced by ULD of one cytokine at a time might be explained by the fact that IL-1β and TNF-α are strongly linked by an autocrine positive loop in monocytes after LPS exposure [89]. Taken together, these results support the rationale for the 2LARTH^®^ formula, which was developed to reduce the symptoms of rheumatic pathologies including RA.

## 7. Micro-Immunotherapy: A Multiple Immune-Targeted Ultra-Low-Dose-Based Strategy to Calm Chronic Inflammation in RA

One of the main challenges of immunotherapy lies in the feasibility of the immune system management by a proper targeting of the immunopathological activity without further affecting the immunosurveillance. Thus, by combining both the advantages of using the immune system players themselves and extremely low dosages, MI could be at a crossroads in the RA treatment too, as the side effects of a conventional treatment would be reduced and/or avoided.

The 2LARTH^®^ is a medicine that was developed to attenuate the symptoms of rheumatic diseases including RA and which is currently notified as a homeopathic medicine under notification number 1507CH36 F1 by the Federal Agency for Medicines and Health Products in Belgium. This medicine consists of different capsules, each one intended to be taken according to the order in which they are packaged in the blister (from 1 to 10), to give daily information to the body. All the capsules are composed of the same cytokines/factors at various ULD depending on the capsule number. Of note, the lactose-sucrose pillules are impregnated with IL-1β at either 10 or 17 CH (i.e., 10–17 CH), TNF-α (10–17 CH), IL-2 (10–12 CH), SNA^®^-HLA-I (10–16 CH), SNA^®^-HLA-II (10–16 CH) (conceived to target regions of HLA class I and II genes) and SNA^®^-ARTH (designed to target the human IL-2 gene at 10–16 CH). The rationale of the HLA-I and HLA-II SNA^®^ targeting relies on the association of RA with key major histocompatibility complexes (MHC) belonging either in type I [90,91], or type II [92,93]. Further studies are still needed to strengthen the knowledge about the effect of these SNA^®^ in the context of RA. The formulation of each capsule of the 2LARTH^®^ is summarized in Table 2.

In the same manner as for the evaluation of the anti-inflammatory effects of one single cytokine at a time, IL-1β or TNF-α at 27 CH, Floris et al. reported that 2LARTH^®^ has a significant impact in reducing IL-1β secretion in human enriched monocytes stimulated with LPS [94]. Such effect was noticed at lactose-sucrose concentrations ranging from 5.5 to 22 mM, compared to both untreated and vehicle-LPS stimulated cells. Moreover, the secreted level of TNF-α was also reduced at concentrations ranging from 2.25 to 22 mM in the same conditions, as well as the IL-6 secretion, at 11 and 22 mM. Furthermore, the effects of the treatment have been evaluated in vivo, in a CIA mice model, in which a daily treatment respecting the sequential order of 2LARTH^®^ was administered by oral gavage, starting 30 days after the first immunization [95]. Results showed its efficacy in reducing the clinical score, the degree of edema and the inflammation in treated animals, compared with the control ones. Histological analysis revealed that the synovial hyperplasia was markedly decreased in MI-treated mice, compared to the vehicle group, and the circulating levels of TNF-α decreased too, and were comparable to the healthy control ones.

The “three birds with one stone” strategy of 2LARTH^®^ is illustrated in Figure 3B, as IL-1β, TNF-α, and IL-2 are the principal therapeutic targets, all three used at ULD. We have widely discussed the first two targets, and less dwelt about the third.

Little is known about the genetic background of IL-2 in RA, but it was found that specific diplotypes, meaning the combination of haplotypes from both parents, in occurrence, the IL-2 TG:GG was positively associated with RA in the Turkish population [96]. Interestingly, the implication of this cytokine in joint-related pathologies seems to be dual and its use as a therapeutic target may be approached in a modulatory manner as contradictory data were addressed regarding this cytokine in this particular context. For instance, Paradowska-Gorycka et al. found that patients affected with RA or osteoarthritis have higher serum levels of IL-2 than the healthy controls [97], whereas Wang et al. reported an opposite trend, as the serum level of IL-2 was downregulated in RA patients group compared with healthy one [98].

Beyond the previously discussed mutational background of IL-1β, TNF-α and IL-2 which may predispose to RA development, many epigenetic events also contribute to the fine-tuned regulation of these factors. The plasticity of all these processes ultimately lead to a dynamic panel of pro-inflammatory mediators, highly susceptible to changes over time. A study focused on deciphering if the cytokine profile found in RA patients serum changed over the course of the disease showed that, 3 months after the initial diagnosis, IL-1β was positively correlated with two of the acute phase inflammatory reactants: CRP (C reactive protein) (R^2^ = 0.642) and ESR (erythrocyte sedimentation rate) (R^2^ = 0.579) [99]. Interestingly, this study also reported that: (i) the levels of IL-1β and IL-2 were both positively correlated (R^2^ = 0.829) 6 months post-diagnosis, (ii) IL-1β and TNF levels were not only correlated at the time of the diagnosis (R^2^ = 0.956) but also 6 months later (R^2^ = 0.847), (iii) IL-2 and TNF showed a positive correlation (R^2^ = 0.738) 6 months post-diagnosis too. This study conducted in Poland would need to be generalized to more patients, but it reinforces the fact that a holistic approach to RA treatment, with multiple cytokine targets, could be highly beneficial for patients. These results are in line with the fact that both IL-2 and TNF-α were found to be parts of a specific pro-inflammatory cytokine profile also defined by high levels of CXCL10 and IL-6, characterizing severe outcomes in systemic autoimmune diseases [100].

The wide therapeutic potential of IL-2 in many autoimmune and inflammatory diseases can be illustrated by the fact that, when the dose is high, IL-2 was reported to promote the proliferation of effector T cells, while, when the dose is low, it allowed an activation of the Tregs [101]. In addition, the effect of low dose IL-2 (5 IU/mL) was also shown to inhibit osteoclastogenesis in vitro, a mechanism which is implicated in the bone degradation also occurring in RA [81]. Furthermore, in their study, Yang et al. used the TNF-α-stimulated MH7A cells to show that this stimulation induces both NO and IL-2 production in this “fibroblast-like” synoviocytes, thus reinforcing the modulatory loop existing between TNF-α and IL-2 [102]. As the rationale of using IL-2 in the 2LARTH^®^ is based on its immunomodulatory functions, the 10–12 CH dilution range employed in the sequence of the medicine allows the body’s responses to freely adjust by themselves, depending on the organism-specific needs at a particular time (Figure 3B). Moreover, the SNA^®^-ARTH, which is especially designed to directly target the IL-2 gene, might reinforce and act synergistically with the ULD IL-2.

More preclinical studies are still needed to understand its mode of action, the synergy of its multiple regulators, the benefits of its sequential strategy, and clinical studies are indispensable to confirm and bring out the potential benefit of MI in the context of RA treatment.

## 8. From the Mouth to the Body: How ULD-Based Medicines Might Act

As the MI delivery route is oromucosal, and the ULD concept is based on a global vision of the body and its internal balance, the crosstalk between the sublingual mucosa cells, the gut tract, and the immune system is a leverage on which this kind of therapy ultimately operates with in order to maintain one’s organism inner equilibrium. Moreover, oral immune therapy, as a method of immune systemic modulation, uses the inherited capabilities of the innate system of the gut to readdress both the innate and adaptive immune responses [103]. In line with these considerations, the relationship between TNF-α and the gut epithelium was assessed in transgenic Rag1 mice lacking adaptive T and B cells, in which it was reported that this factor induced enhanced apoptosis in the epithelial cells from the duodenum [104]. In addition, an interesting study correlates the TNF-α serum levels of RA patients with their intestine microbiome [105]. The results showed that *Pelagibacterium* was positively correlated with TNF-α, whereas *Oxalobacter* and *Blautia* were negatively correlated with this factor, suggesting that the gut microflora could be involved in RA progression by altering the cytokine levels. Altogether, these pieces of evidence reinforce the fact that the systemic cytokine modulation occurring in RA could be perpetuated by several integrated factors and that RA treatment could legitimately be considered in a holistic approach, in which the MI has its place.

At cellular levels, MI could be explained by the hormetic dose-specific responses curve characterized by its non-linear shape, when the “stimulus” is tested in a wide range of concentrations. The “hormesis” concept may underpin the cellular adaptation to these ultra-low cell responses, as this notion has been applied to a broad spectrum of independent cellular functions such as proliferation, DNA repair, antioxidant response or autophagy for instance [106]. Hormesis thus characterizes the cellular responses induced by extremely low levels of potentially toxic agents, as adaptive responses of biological systems, in order to improve their functionality and/or tolerance to severe environmental challenges. Hormetic responses are evolutionary-conserved, highly generalizable across phyla and thought to result from multiple integrative signal-transduction processes, thus coordinating a final holistic response. Importantly, hormetic signals originating from a stressed tissue can be transferred to distant tissues or organs, as an overall phenomenon called “remote conditioning” [107]. These manifestations of hormesis have been documented in the context of cardioprotective strategies establishment and pinpointed the remote conditioning as exactly as a systemic response, where (1) the stimulus (chemical substances, electrical signal), (2) the transfer (neuronal and humoral mediators), and (3) the target (receptor activations, transduction pathways within the heart cells) levels were taken into consideration. As RA is also a systemic disease, the hormesis and especially its remote conditioning component could be employed as an explanation of the effectiveness of the ULD used in MI.

## 9. Conclusions

**Figure 4:**
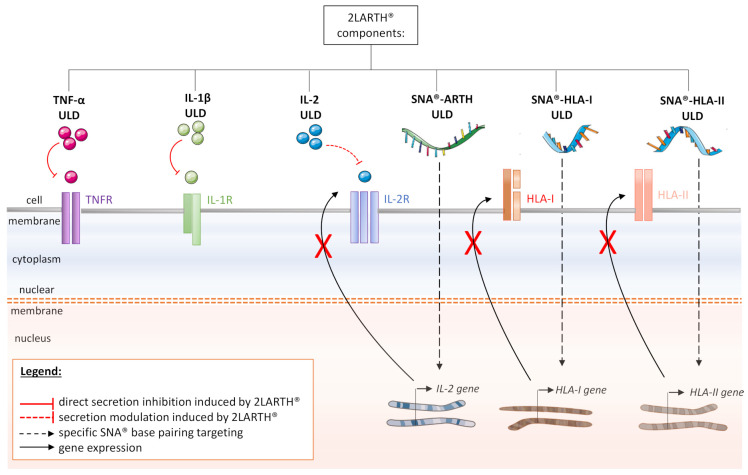
Schematic representation of the multi-targets actions of 2LARTH^®^ within the cells of the synovial niche in rheumatoid arthritis (RA): HLA: Human leukocyte antigen; IL-1R: Interleukin-1 receptor; IL-2R: Interleukin-2 receptor; SNA^®^: Specific nucleic acid; TNFR: TNF receptor; ULD: Ultra-low dose.

In conclusion, TNF-α and IL-1β are two key factors involved in the onset and spreading of RA and their increased levels maintain/sustain the chronic inflammation observed both at systemic and articular levels. Their therapeutic targeting is a useful and efficient strategy, as it allows to impair the systemic and synovial-niche cellular crosstalk that they elicit, and which contributes to the pernicious fueling of the pathology. To date, the treatments are still hampered by important side effects and alternative therapies would be of great value for the patients. As a more holistic approach, the MIM claims to restore the body’s homeostasis through the use of multiple immunologic agents at ULD (Figure 4). Compared to the single-target approach, MIM 2LARTH^®^ formulation combines together three therapeutic targets: TNF-α, IL-1β, and IL-2, and its ULD-based strategy is promising because it should exert fewer adverse effects. Even if additional preclinical and clinical data are still needed, MIM might be the way to gently intervene, in order to reestablish inner imbalances and to overcome immune diseases such as RA.

## Figures and Tables

**Table 1:** Non-exhaustive list of miRNAs related to RA pathogenesis and their biological effects on the pro-inflammatory cytokines and factors IL-1β and TNF-α. ND: no data.

miRNA	miRNA Levels in RA	Signaling Pathways/Cytokines Involved or Biological Effects	Authors
126-3-p	up in RA serum	According to the QIAGEN IPA Software, these miRNAs are involved in a network of potential direct and indirect interactions impacting transcription factors and the downstream production of cytokines such as IL-1β and TNF-α.	Cunningham CC et al., 2021 [71]
let-7d-5p	up in RA serum		
431-3p	up in RA serum		
221-3p	up in RA serum		
24-3p	up in RA serum		
130a-3p	up in RA serum		
339-5p	up in RA serum		
let-7i-5p	up in RA serum		
17-5p	down in RA serum		
17-5p	down in erosive RA	MiR-17-5p injections into the paw of arthritic mice induced a reduction of IL-6 and IL-1β, but not TNF-α.	Aurélie Najm et al., 2020 [76]
26b-5p	down in RA serum	MiR-26b-5p mimics treatment alleviated inflammatory responses and reduced Th17 proportion in CIA mice.	Ming-Fei Zhang et al., 2021 [77]
140-5p	ND	Their simultaneous overexpression in Osteoarthritic chondrocytes reduces NF-κb phosphorylation, as well as the expression of TLR4, IL-1β, IL-6 and TNF-α.	Ioanna Papathanasiou et al., 2020 [75]
146a			
140-5p & 140-3p	down in synovial tissues and synovial fibroblasts from RA patients	Intra-articular delivery of these miRNAs ameliorates the clinical and histological features of RA.	Jia-Shiou Peng et al., 2016 [74]
140-5p	ND	The interplay of the miR-140-5p and TNF-α was assessed in a pulmonary arterial hypertension model. The direct targeting of this miRNA towards the TNF-α 3′UTR region was confirmed by Luciferase assays.	Tian-Tian Zhu et al., 2019 [73]
146a	up in CD4^+^ T cells from RA patients	MiR-146a expression is positively correlated with levels of TNF-α. In vitro studies showed that TNF-α upregulated miR-146a expression in T cells.	Jingyi Li et al., 2010 [78]
363	down in CD4^+^ T cells from RA patients		
498	down in CD4^+^ T cells from RA patients		
10a	down in the fibroblast-like synoviocytes of RA patients	MiR-10a downregulation could be triggered by TNF-α and IL-1β and results in the activation of the NF-κB pathway and the promotion of IL-1β, TNF-α, IL-6, IL-8 and MCP-1 expression.	Nan Mu et al., 2016 [79]

**Table 2:** Table summarizing the 2LARTH^®^ formulation and dilutions employed in the different capsules. The dilutions of each cytokine/factor/SNA^®^ at ULD are indicated in CH (Centesimal Hahnemannian). MI medicines are based on the principle that a given immune regulator exerts either stimulatory effects when used at LD or displays modulatory/inhibitory features when prepared at ULD. ULD aim at modulating (from 8 to 12 CH) and lowering (higher than 12 CH) the expression of protein with upregulated levels. HLA: Human Leukocyte Antigen; IL: Interleukin; SNA^®^: Specific Nucleic Acid; TNF-α: Tumor Necrosis Factor-α; ULD: Ultra-Low Dose.

2LARTH^®^		
Ingredients	Modulatory ULD	Inhibitory ULD
IL-1	10	17
IL-2	10	12
TNF-α	10	17
SNA^®^-ARTH	10	16
SNA^®^-HLA-I	10	16
SNA^®^-HLA-II	10	16

## Abbreviations

ACPA	Anti-citrullinated peptide antibodies
ACR	American College of Rheumatology
CCP	Cyclic citrullinated peptides
CH	Centesimal Hahnemannian
CIA	Collagen-induced arthritis
COX-2	Cyclooxygenase-2
CRP	C reactive protein
Cx43	Connexin-43
DMARD	Disease-modifying anti-rheumatic drug
ESR	Erythrocyte sedimentation rate
FDA	Food and Drug Administration
FDOJ	Fatty-degenerative osteolysis of the jawbone
GM-CSF	Granulocyte-macrophage colony-stimulating factor
HAQ	Health Assessment Questionnaire
HLA	Human leukocyte antigen
IFN-γ	Interferon-γ
IκB	Inhibitor of nuclear factor-κB
IL-1β	Interleukin-1β
JAK3	Janus-activated kinase 3
LD	Low dose
LDA	Low disease activity
LOX	Lipoxygenase
LPS	Lipopolysaccharide
MAPK	Mitogen-activated protein kinase
MCC	Merkel cell carcinoma
MCPyV	Merkel cell polyomavirus
MHC	Major histocompatibility complex
MI	Micro-immunotherapy
MCP1	Monocyte chemoattractant protein-1
MIM	Micro-immunotherapy medicine
MIP1α	Macrophage inflammatory protein-1 alpha
MMP	Matrix metalloproteinase
MyD88	Myeloid and differentiation primary response 88
NF-κB	Nuclear factor-κB
PBMC	Peripheral blood mononuclear cell
PEG	Polyethylene glycol
PGE2	Prostaglandin E2
RA	Rheumatoid arthritis
RANKL	Receptor activator of nuclear factor-κB ligand
RANTES	Regulated upon activation, normal T cells expressed and secreted
SKA	Sequential kinetic activation
SNA^®^	Specific nucleic acids
TIR	Toll-IL-1 receptor
TNF-α	Tumor necrosis factor-α
TNFR1	TNF receptor type 1
TRAF1-C5	TNF receptor-associated factor 1 and complement component-5
ULD	Ultra-low dose
VEGF	Vascular endothelial growth factor
